# Deletion of a 4977-bp Fragment in the Mitochondrial Genome Is Associated with Mitochondrial Disease Severity

**DOI:** 10.1371/journal.pone.0128624

**Published:** 2015-05-29

**Authors:** Yanchun Zhang, Yinan Ma, Dingfang Bu, Hui Liu, Changyu Xia, Ying Zhang, Sainan Zhu, Hong Pan, Pei Pei, Xuefei Zheng, Songtao Wang, Yufeng Xu, Yu Qi

**Affiliations:** 1 Department of Central Laboratory, Peking University First Hospital, No. 8, West District, Beijing, 100034, China; 2 Department of Respiratory, Beijing Children’s Hospital, Beijing, 100045, China; 3 Department of Clinical Laboratory, Peking University First Hospital, No. 8, West District, Beijing, 100034, China; 4 Department of Biostatistics, Peking University First Hospital, No. 8, West District, Beijing, 100034, China; University of Texas Health Science Center at San Antonio, UNITED STATES

## Abstract

Large deletions in mitochondrial DNA (mtDNA) may be involved in the pathogenesis of mitochondrial disease. In this study, we investigated the relationship between a 4,977-bp deletion in the mitochondrial genome (ΔmtDNA^4977^) and the severity of clinical symptoms in patients with mitochondrial disease lacking known point mutations. A total of 160 patients with mitochondrial disease and 101 healthy controls were recruited for this study. The copy numbers of ΔmtDNA^4977^ and wild-type mtDNA were determined by real-time quantitative PCR and analyzed using Spearman’s bivariate correlation analysis, t-tests, or one-way ANOVA. The overall ΔmtDNA^4977^ copy number per cell and the proportion of mtDNA^4977^ relative to the total wild-type mtDNA, increased with patient age and symptom severity. Surprisingly, the total mtDNA copy number decreased with increasing symptom severity. Our analyses revealed that increases in the proportion and total copy number of ΔmtDNA^4977^ in the blood may be associated with disease severity in patients with mitochondrial dysfunction.

## Introduction

Mitochondria play important roles in cellular energy metabolism and the generation of free oxygen radicals. Each mitochondrion contains 2–10 copies of circular mitochondrial DNA (mtDNA) containing many essential genes. More than 130 different mtDNA deletions have been reported in human populations [1], and larger deletions are often associated with mitochondrial diseases, including chronic progressive ophthalmoplegia [2], Kearns-Sayre syndrome [3], Pearson’s syndrome [4], maternally inherited deafness, and adult-onset diabetes [5]. A 4,977-bp mtDNA deletion (ΔmtDNA^4977^) located between nucleotides 8,469 and 13,447 has been reported in various diseases and accumulates with age [6–7]. This fragment includes the genes encoding ATPase6, ATPase8, cytochrome oxidase III, NADH dehydrogenase subunit 3 (ND3), ND4, ND4 subunit L (ND4L), and ND5. Therefore, cells harboring ΔmtDNA^4977^ lack several vital oxidative phosphorylation (OXPHOS) genes leading to an overall decrease in energy supply, particularly in the brain and muscles. Previous reports have focused on associations between point mutations and mitochondrial disease pathogenesis, rather than associations involving larger deletions. Moreover, large population-based studies on the role of ΔmtDNA^4977^are rare, and its function in mitochondrial disease pathogenesis remains heavily disputed [8–14]. One of the critical factors determining the mitochondrial phenotype is the copy number ratio of mutant to wild-type (WT) mtDNA. We previously reported that WT mtDNA copy number is a better predictor of disease severity in patients with mitochondrial encephalomyopathy, lactic acidosis, and stroke-like episodes (MELAS) syndrome that harbor A3243G mutations in their mitochondrial genome [15]. In this study, we evaluated the relationship between ΔmtDNA^4977^ copy number in the blood and the severity of clinical symptoms in patients with mitochondrial disease.

## Materials and Methods

### Patients

A total of 160 patients with mitochondrial disease lacking mutations with known disease associations (i.e., A3243G, A8344G, T8993C, T8993G, A1555G, G11778A, and G13513A) were recruited from the Departments of Pediatrics and Neurology, Peking University First Hospital from December 2003 to December 2013. The average age of the patient population was 4.6 years (range: 0.1–20 years). Clinical diagnoses were based on the major and minor diagnostic criteria of mitochondrial diseases proposed by Bernier [16] and Rodenburg [17]. In the absence of a genetic diagnosis, biochemical findings such as abnormalities in lactic acid levels, enzyme activity, and muscle biopsies were used for diagnosis. Clinical manifestations included seizure, stunting, developmental regression, ataxia, myopathy, lactic acidosis, vomiting, diarrhea, constipation, hearing loss, vision loss, mental or psychological problems, arrhythmia, and diabetes mellitus, amongst others, and the manifestations were categorized as mild, moderate, and severe based on the Newcastle Paediatric Mitochondrial Disease Scale (NPMDS). Disease severity was evaluated based on the extent of organ involvement. In addition, 101 healthy subjects with an average age of 6.7 years (range: 0.1–20 years) were recruited as normal controls from the Physical Examination Center of Peking University First Hospital. Physical and biochemical examinations were performed to exclude systemic diseases, including neurological diseases and diabetes mellitus. Patients and normal controls were divided based on age into a younger group (<10 years of age) and an older group (10–20 years of age). Written informed consent was obtained from both patients and controls or their guardians and this study was approved by the Medical Ethics Committee of Peking University First Hospital.

### mtDNA copy number analysis

Total DNA was extracted from peripheral blood leukocytes and mtDNA-depleted ρ^0^ cells using Miller’s method [18]. DNA from ρ^0^ cells was used as a blank control in PCR reactions to examine mtDNA copy number. The primers used to quantify the proportion of mtDNA copies harboring the 4,977-bp deletion were as follows [14]: 5′-AAAATATTAAACACAAACTACC ACCTACCTCCCTCACCAT (forward primer, nucleotides 8,445–8,483), 5′-GGGGAAGCGA GGTTGACCTG (reverse primer, nucleotides 13,632–13,651), and 5′-FAM-TGGCAGCCTAG CATTAGCAGG-TAMRA (TaqMan probe [19], nucleotides 13,462–13,482). Primers for the mitochondrial *ND1* gene used to quantify the mtDNA copy number were as follows: 5′-ATTCGATGTTGAAGCCTGAGACT (forward primer, nucleotides 3,928–3,950), 5′-TGACCCTTGGCCATAATATGATT (reverse primer, nucleotides 3,842–3,864), and 5′-Hex-TTCGGACTCCCCTTCGGCAAGG-BHQ1 (TaqMan probe, nucleotides 3904–3925). Primers for the single-copy nuclear gene *HBB* (β-hemoglobin) used to measure nuclear genomic DNA copy number were as follows: 5′-ACCTCAAGGGCACCTTTGC (forward primer), 5′-AAAACATCAAGCGTCCCATAGAC (reverse primer), and 5′-FAM-CACTGTGACAAG CTGCACGTGGATCC-BHQ1 (TaqMan probe). Three recombinant plasmids containing fragments of the *ND1*, ΔmtDNA^4977^, and *HBB* were constructed for use as copy number standards. Quantitative PCR reactions were run using an ABI 7500 instrument with thermal cycling conditions of 95°C for 10 min, followed by 40 cycles of 95°C for 15 sec and 60°C for 60 sec. The establishment of copy number standards and assay methods were described in detail previously [15]. Given that one cell contains two copies of the *HBB* gene, the total copy numbers of mtDNA and ΔmtDNA4977 per cell could be calculated. The average proportion of ΔmtDNA^4977^ in cells was derived from the ratio of the ΔmtDNA^4977^ copy number per cell the total mtDNA copy number per cell. ΔmtDNA^4977^ copy numbers per cell and total mtDNA copy number per cell were converted into normally distributed logarithmic values.

### Statistical analysis

All statistical analyses were implemented in SPSS 17.0 (IBM Software, Armonk, NY, USA). Numerical values are presented as means ± standard deviations (SD). The total mtDNA copy number per cell, ΔmtDNA^4977^ copy number per cell, and proportion of ΔmtDNA^4977^ were compared between mitochondrial disease patients and normal controls using *t*-tests. One-way ANOVA and Bonferroni tests were used to compare the total mtDNA copy number per cell, ΔmtDNA^4977^ copy number per cell, and proportion of ΔmtDNA^4977^ among disease severity groups. Correlations between observational indices and disease severity or clinical symptoms were analyzed using Spearman’s correlation. Only clinical features with a frequency of >10% were analyzed. A *p*-value of less than 0.05 was considered statistically significant.

## Results

### ΔmtDNA^4977^ copy number accumulates with age

The proportion of ΔmtDNA^4977^ has been reported to accumulate with age [7]. To determine if this trend occurred in our patient population, mtDNA was harvested from peripheral blood leukocytes and the ratio of ΔmtDNA^4977^ to total mtDNA per cell was assessed. In normal controls, the older patient group (10–20 years old) exhibited a higher ΔmtDNA^4977^ copy number per cell (2.60 ± 0.41 vs. 1.70 ± 0.65 per 10^4^ cells; *p* < 0.001), total mtDNA copy number per cell (2.21 ± 0.46 vs. 1.76 ± 0.51; *p* < 0.001), and ratio of ΔmtDNA^4977^ to total mtDNA (2.36 ± 0.50 vs. 1.86 ± 0.65, respectively, *p* < 0.001) than those of the younger group (0–10 years old). For patients with mitochondrial disease, older patients also had a higher ΔmtDNA^4977^ copy number per 10^4^ cells (2.97 ± 0.48 vs. 2.79 ± 0.50; *p* < 0.01), and ΔmtDNA^4977^ ratio (3.09 ± 0.74 vs. 2.66 ± 0.63; *p* < 0.01) than their younger counterparts; however, the total mtDNA copy number per cell was not significantly different between the older and younger patients (1.96 ± 0.50 vs. 2.07 ± 0.39; *p* <0.05) (Table 1).

**Table 1:** Differences in total mtDNA copy number, ΔmtDNA^4977^ copy number, and the ratio ΔmtDNA^4977^ to total mtDNA in patients with mitochondrial disease (MCD) and healthy controls.

	0 < age (years) < 10				10 ≤ age (years) < 20			
	MCD	Controls	*t*	*p*	MCD	Controls	*t*	*p*
N	135	70			25	31		
ΔmtDNA^4977^ copy number /10^4^cells*	2.79 ± 0.50	1.70 ± 0.65	4.50	<0.001	2.97 ± 0.48	2.60 ± 0.41	-3.67	<0.01
total mtDNA copy number /cell*	2.07 ± 0.39	1.76 ± 0.51	12.26	<0.001	1.96 ± 0.50	2.21 ± 0.46	3.13	<0.01
proportion of ΔmtDNA^4977^ (%)**	0.12 ± 0.19	0.02 ± 0.03	5.85	<0.001	0.37 ± 0.54	0.04 ± 0.05	2.98	<0.001

*Data are presented as (log) means ± SD,

**Data are presented as means ± SD.

### Increased proportion of ΔmtDNA^4977^ is correlated with disease severity

**Fig 1:**
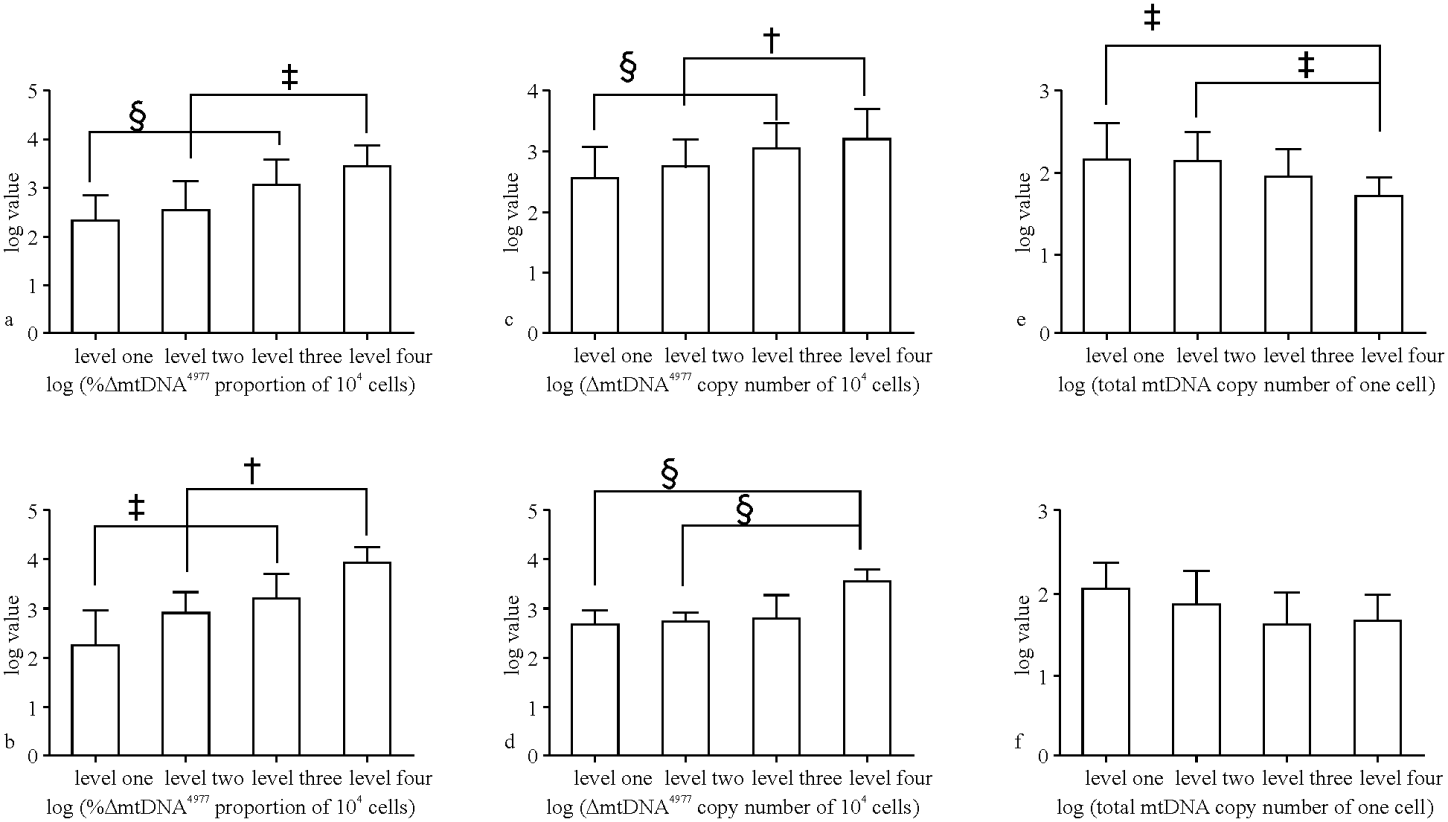
Associations between ΔmtDNA^4977^ copy number or proportion and symptom severity in patients with mitochondrial disease. Panels a, c, and e indicate the younger group (<10 years old) and panels b, d, and f indicate the older group (10–20 years old). †: p < 0.05; ‡: p < 0.01; §: p < 0.001.

**Fig 2:**
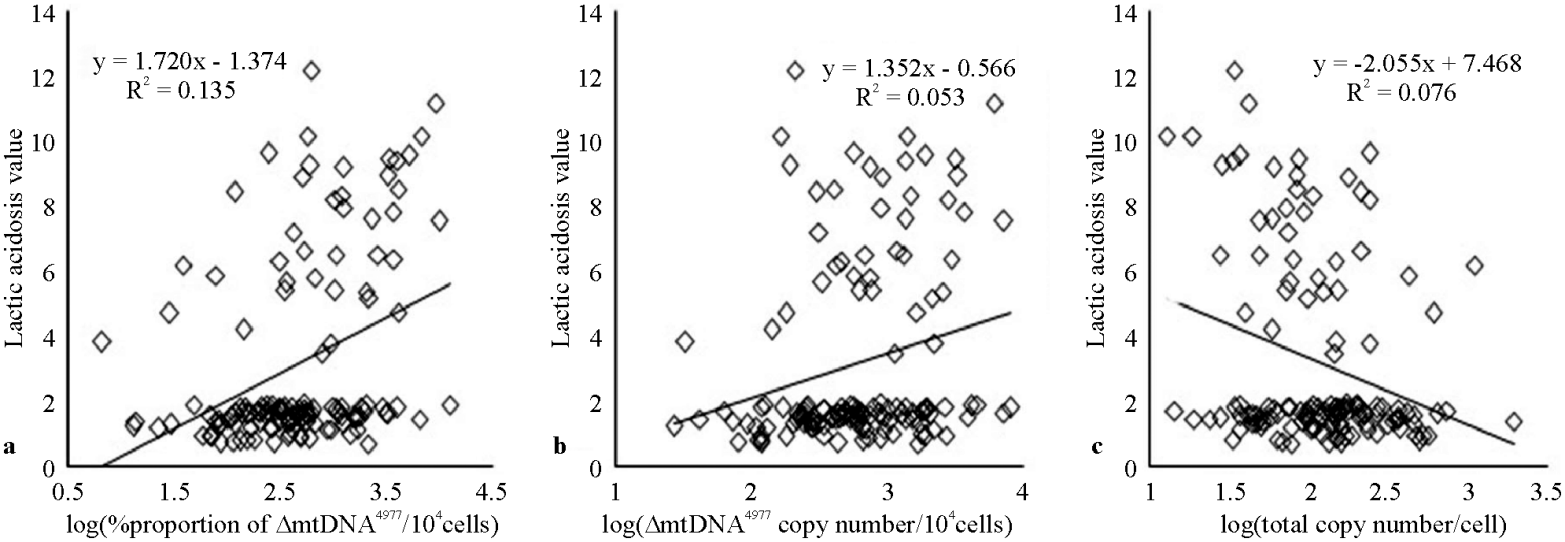
The correlation between lactic acidosis and proportion of ΔmtDNA^4977^ in 10^4^cells, ΔmtDNA^4977^ copy number/10^4^ cells, and total mtDNA copy number/cell in younger mitochondrial disease patients.

We sought to determine whether ΔmtDNA^4977^ increased with the severity of mitochondrial disease symptoms. Notably, the proportion of ΔmtDNA^4977^ was significantly higher in patients with mitochondrial disease than in healthy controls (Table 1). The major clinical manifestations of mitochondrial diseases in the younger patient group included seizure (73.3%), myopathy (36.3%), lactic acidosis (29.6%), stunting (22.2%), developmental regression (21.5%), and mental or psychological symptoms (10.4%), whereas the primary manifestations in the older group were seizure (68.0%), myopathy (52.0%), lactic acidosis (32.0%), vision loss (28.0%), stunting (20.0%), developmental regression (20.0%), mental or psychological symptoms (12.0%), and vomiting/diarrhea/constipation (12.0%) (Table 2). We defined disease severity as 1, 2, 3, or ≥4 based on the number of organs or organ systems involved. These data suggested that the proportion of ΔmtDNA^4977^ increased with disease severity (Fig 1a and 1b). Moreover, the proportion of ΔmtDNA^4977^ was positively correlated with developmental regression (*r* = 0.28, *p* < 0.01) and lactic acidosis (*r* = 0.37, *p* < 0.01, Fig 2a) in the younger group, and mental or psychological symptoms (*r* = 0.41, *p* = 0.04) and vomiting/diarrhea/constipation (*r* = 0.46, *p* = 0.02) in older patients. Furthermore, the proportion of ΔmtDNA^4977^ was positively and strongly correlated with disease severity in both groups (*r* = 0.52, *p* < 0.001 and *r* = 0.77, *p* < 0.001 for younger and older patients, respectively; Table 3), indicating that patients with a higher proportion of ΔmtDNA^4977^ exhibited increased symptom severity.

**Table 2:** Clinical manifestation frequencies in younger and older patients of mitochondrial diseases.

Involved organ	Clinical manifestation	Severity ^a^			Total frequency (%) ^a^
		Mild	Moderate	Severe	
Muscle	Myopathy	13/5	21/4	15/4	36.3/52.0
Renal	Renal function tests	4/1	3/1	0/0	5.2/8.0
Gastrointestinal	Unexplained vomiting/ diarrhea/ constipation	5/2	4/1	1/0	7.4/12.0
Liver	Liver function tests	3/2	1/0	0/0	3.0/8.0
Cardiovascular	ECG changed/ Arrhythmia	5/0	3/2	0/0	5.9/8.0
Endocrine	Blood glucose	6/0	5/2	0/0	8.1/8.0
Respiratory	Respiratory pattern	4/1	3/1	1/0	5.9/8.0
Eye	Vision	1/1	2/3	4/3	5.2/28.0
	Ptosis and eye movement	3/1	4/1	2/1	6.7/12.0
Ear	Hearing	1/0	0/0	1/2	1.5/8.0
Blood	Anemia /Pancytopenia	3/2	2/0	0/0	3.7/8.0
Brain	Seizure	26/4	32/8	41/5	73.3/68.0
	Development regression ^b^				21.5/20.0
	Globally development delay (including growth)	5/0	6/2	19/3	22.2/20.0
	Ataxia ^b^				5.9/8.0
	Mental or psychological problems ^b^				10.4/12.0
other	Lactic acidosis ^b^				29.6/32.0

^a^: The results for the younger and older patient groups are indicated before and after the “/” symbol, respectively.

^b^: The manifestations did not contribute to severity determination.

**Table 3:** The correlation between disease severity and ΔmtDNA^4977^ copy number/10^4^ cells, total mtDNA copy number/cell, and proportion of ΔmtDNA^4977^ in cells in mitochondrial disease patients.

	0 < age (years) < 10		10 ≤ age (years) < 20	
	*r*	*p*	*r*	*p*
log(ΔmtDNA^4977^ copy number/10^4^ cells)	0.39	<0.001	0.61	<0.01
log(total mtDNA copy number/cell)	-0.26	<0.01	-0.43	<0.05
proportion of ΔmtDNA^4977^(%)	0.52	<0.001	0.77	<0.001

### ΔmtDNA^4977^ copy number is correlated with disease severity

Based on the previous results, we examined whether ΔmtDNA^4977^ copy number was itself associated with disease severity. The ΔmtDNA^4977^ copy number per 10^4^ cells was significantly higher in patients with mitochondrial disease than in healthy controls for both age groups (Table 1). In addition, the ΔmtDNA^4977^ copy number per 10^4^ cells was positively correlated with disease severity in both patient groups (Fig 1c and 1d; *r* = 0.39, *p* < 0.001 and *r* = 0.61, *p* <0.01 for the younger and older patient groups, respectively; Table 3). Interestingly, the ΔmtDNA^4977^ copy number per 10^4^ cells was also positively correlated with lactic acidosis (*r* = 0.23, *p* < 0.01, Fig 2b) in younger, but not older patients.

### Increased total mtDNA copy number attenuates disease severity

Based on these analyses, we determined that the total mtDNA copy number per cell was higher in the younger group than in the older group and was inversely correlated to disease severity (Table 1; Fig 1e and 1f). Additionally, the total mtDNA copy number per cell was negatively correlated with disease severity (Spearman’s correlation analysis; *r* = -0.26, *p* <0.01 and *r* = -0.43, *p* <0.05 for the younger and older patient groups, respectively; Table 3). In the younger group, the total mtDNA copy number per cell was also negatively correlated with lactic acidosis (*r* = -0.28, *p* <0.01, Fig 2c) and developmental regression (*r* = -0.22, *p* <0.05), but was negatively correlated with vomiting/diarrhea/constipation in older patients (*r* = -0.44, *p* <0.05).

### Influence of ΔmtDNA^4977^ is more pronounced in patients with symptoms involving three or more organs

In the younger group, the ΔmtDNA^4977^ copy number per 10^4^ cells (F = 5.46, *p* <0.01), total mtDNA copy number per cell (F = 8.62, *p* < 0.001), and proportion of ΔmtDNA^4977^ (F = 16.9, *p* < 0.001) were all significantly different between the four levels of disease severity, except between levels 1 and 2 or 3 and 4 (Fig 1). Accordingly, we pooled patients from groups 1–2 and 3–4 into two groups. Notably, both ΔmtDNA^4977^ copy number per 10^4^ cells (F = 4.86, *p* <0.05) and the proportion of ΔmtDNA^4977^ (F = 9.35, *p* < 0.001) were significantly different among the four levels of disease severity, but the total mtDNA copy number per cell was not significantly different among groups in older patients (F = 2.08, *p* <0.05). After pooling the patient groups, we found that these parameters correlate with disease severity, as seen in Table 4, which presents the two groups after pooling, compared based on *t*-tests.

**Table 4:** After pooling two levels of disease severity, differences in total mtDNA copy number, ΔmtDNA^4977^ copy number, and the ratio ΔmtDNA^4977^ to total mtDNA are shown.

Disease severity levels	0 < age (years) < 10			10 ≤ age (years) < 20		
	1–2	3–4	*p*	1–2	3–4	*p*
N	92	43		12	13	
log(ΔmtDNA^4977^ copy number/10^4^cells)	2.66 ± 0.48	3.06 ± 0.43	<0.001	2.73 ± 0.22	3.19 ± 0.55	<0.05
log(total copy number/cell)	2.15 ± 0.39	1.91 ± 0.35	<0.01	1.95 ± 0.34	1.63 ± 0.36	<0.05
proportion of ΔmtDNA^4977^ (%)	0.06 ± 0.08	0.24 ± 0.28	<0.001	0.08 ± 0.08	0.59 ± 0.64	<0.001

## Discussion

Mitochondrial DNA is continuously exposed to oxidative stress, and is therefore more easily mutated than nuclear DNA. Thus far, most known mitochondrial DNA mutations are point mutations and deletions. The ΔmtDNA^4977^ deletion is the most common large deletion in the mitochondrial genome. In some previous studies, ΔmtDNA^4977^ was not detected in whole blood [20, 21]; however, we found that the proportion of ΔmtDNA^4977^ was 0.00001–1.9% in patients with mitochondrial disease and healthy controls under 20 years of age. Assuming an upper limit of 95% for the normal proportion ΔmtDNA^4977^, we detected an abnormally high proportion of ΔmtDNA^4977^ in 32.6% (44/135) of younger patients and 32% (8/25) of older patients with mitochondrial disease. This is consistent with the results of a previous study [22]. Healthy infants and children were also found to have this deletion [23]. Although the proportion of ΔmtDNA^4977^ was only moderately high in our study, the difference between patients and normal controls was statistically significant (p < 0.05), emphasizing the importance of ΔmtDNA^4977^ in mitochondrial disease pathogenesis. Additionally, slight differences were detected in the proportion of ΔmtDNA^4977^ among different disease severity groups, but the clinical manifestations were higher, indicating that these differences were important to mitochondrial disease.

The finding that ΔmtDNA^4977^ accumulates with age remains controversial. Von Wurmb N et al. reported that this deletion accumulates with age [24], and others have reported that it is related to the occurrence of various types of degenerative diseases and aging [25,26]. However, other studies have found that this mutation is not age-dependent or the relationship between age and ΔmtDNA^4977^ proportion was inconclusive [21]. In the present study, we found that ΔmtDNA^4977^ copy number and the ratio of ΔmtDNA^4977^to total mtDNA were related to patient age. As such, we divided patients into two groups based on age, similar to the groups used in other studies [27]. Considering that the proportion of ΔmtDNA4977 was different between patients with mitochondrial disease and healthy controls, we suspect that ΔmtDNA^4977^ may accumulate from birth or earlier and likely plays a role in the development of mitochondrial disease.

It remains unclear why there is no consistent relationship between age and ΔmtDNA^4977^, but the current literature suggests that these discrepancies may result from experimental differences related to the patient population or methodology.

In our study, the relative proportion of ΔmtDNA^4977^ was the most important factor correlated with disease severity. Patients exhibiting symptoms involving ≥3 organs had a higher proportion of ΔmtDNA^4977^. Moreover, the proportion of ΔmtDNA^4977^ was positively correlated with clinical symptoms. Interestingly, the total mtDNA copy number per cell was higher in younger patients and lower in older patients when compared to age-matched healthy controls. A similar result was reported in studies of unfertilized oocytes [28]. Furthermore, Rao et al. found that the total mtDNA copy number tended to be higher in hemodialysis patients positive for ΔmtDNA^4977^ [29]. This phenomenon in younger patients may be caused by a compensatory effect [30]. Mitochondria can bear up to 90% damaged mtDNA in the case of WT mtDNA supplementation [31]. To attain normal mitochondrial function, the organism increases the total mtDNA copy number with the goal of maintaining WT mtDNA copy number, particularly in organs with high oxygen demand, such as the brain and muscle. When this compensatory effect is compromised, the WT mtDNA copy number declines, and patients experience more severe clinical symptoms. In the mitochondrial disease group, the total mtDNA copy number decreased with advanced age, and this may be due to the “replication advantage” [32]. Specifically, deletion fragments are smaller than wild-type mtDNA fragments, and smaller molecules replicate faster than larger molecules. This phenomenon could result in a reduction of the total mtDNA copy number. In the healthy population, the total mtDNA copy number increased with age in patients ranging from 10–20 years old. Body height, weight, cognitive and physical abilities [33], and stress-response ability increase during this period, causing increased energy demand. While the precise mechanism regulating mtDNA copy number is unclear, low mtDNA copy number may stimulate the expression of mitochondrial genes in older people [34, 35].

Lactic acidosis is an important clinical laboratory abnormality in mitochondrial patients. Munnich [36] and Zhang [37] studied adult and child patients with mitochondrial disease and found lactic acidosis in >50% patients. In our study, lactic acidosis was found in approximately 30% of patients, which is similar to the results of a previous study of 235 children with mitochondrial diseases [38], and may represent the true prevalence of lactic acidosis in children [39]. We found that the ΔmtDNA^4977^ proportion and copy number as well as the total mtDNA copy number were all correlated with lactic acidosis in the younger group, likely owing to the effects of this deletion on the lactate production pathway; however, this finding remains to be confirmed experimentally.

The mechanism underlying the induction of mitochondrial diseases by ΔmtDNA^4977^ remains unknown. Mitochondria play a crucial role in reactive oxidative species (ROS) production, a known pathogenic factor. Mitochondrial mutations inhibit OXPHOS and the electron transport chain, increase the level of oxidative stress, and result in ROS accumulation. As such, these increased ROS concentrations are more likely to injure mitochondrial genes than nuclear genes and thus affect mtDNA replication. In addition, the 4,977-bp mtDNA sequence had a 13-bp duplicate sequence (5’-ACCTCCCTCACCA), the edge of which is a direct repeat. Additionally, the nearby region where the enzyme protein is located was AT-rich and easily damaged by oxidative stress. Therefore, compared with other mutations, it is more prone to cause mitochondrial disease. ΔmtDNA^4977^ in the loss of many genes related to OXPHOS and interferes with energy metabolism. The WT mtDNA copy number decreased in association with an increase in the ΔmtDNA^4977^ copy number per cell and a decrease in the total mtDNA copy number per cell. In our study, we found that the ΔmtDNA^4977^ copy number facilitated the progression of mitochondrial disease, but seemed to attenuate seizure and myopathy. Therefore, the deletion may not be the primary cause but a related factor, together with other genetic abnormalities lead to the emergence of clinical manifestations in patients with mitochondrial disease.
